# Channel management in virtual care

**DOI:** 10.1038/s41746-020-0252-4

**Published:** 2020-03-25

**Authors:** Matt Desruisseaux, Vess Stamenova, R. Sacha Bhatia, Onil Bhattacharyya

**Affiliations:** 1 McGill University, Montreal, QC Canada; 2 Women’s College Hospital Institute for Health Systems Solutions and Virtual Care, University of Toronto, Toronto, ON Canada; 3 University Health Network, Toronto, ON Canada; 4 Department of Family and Community Medicine, University of Toronto, Toronto, ON Canada

**Keywords:** Health policy, Health care economics, Health services, Technology

## Abstract

Many virtual care initiatives focus heavily on video visits, essentially mimicking face-to-face visits. Meanwhile, clinicians in established settings continue to use the oldest modality, phone calls, and some use the most ubiquitous, asynchronous messaging. The latter, along with live chat and chatbots, could be transformative if workflows were redesigned to incorporate it. With multiple modalities now available for use in virtual care, the central problem is to direct patient-provider interactions to the channels generating the most value. Marketers call this channel management and use sophisticated approaches to implement it. We propose an adaptation of channel management to virtual care and discuss anticipated challenges to its implementation.

## Introduction

Banks did not develop online services by attempting to mimic in-branch encounters. The mobile experience does not begin by videoconferencing with a teller. Instead, banks use apps, websites, call centers, ATMs and branches in distinct combinations across products and customer segments, with a razor-sharp focus on managing these channels. Videoconferencing is only now emerging, specifically for affluent customers and complex products where this high-touch channel is worth the additional cost.

Contrast this with telehealth, where many modalities have been considered and tried, yet what prevails is videoconferencing with one’s physician or with the next available one. These interactions mimic, respectively, the office appointment and the walk-in visit. The province of Ontario, Canada has offered video visits since 2006, yet only recently piloted other modalities^1,2^. Large providers like Teladoc (over 10,000 virtual visits per day)^3^ and American Well, among others, market telehealth primarily as video—via apps, kiosks, hospital carts, electronic medical records, and soon home televisions. One in three Americans have had a video visit^4^, while a similar proportion have had a virtual visit through any channel^5^, suggesting that the vast majority of virtual care interactions in the US occur via video.

To be sure, video can increase access and, at least for patients, generate savings compared to in-person visits^6–8^. But how does it compare to other modalities such as messaging, which has taken over other realms of our lives? Except for a few attempted comparisons of virtual care modalities^9,10^, there is little published evidence and few conceptual frameworks to help answer this question.

## Conceptual foundations

To understand when each channel is most appropriate, we propose the interaction as the unit of analysis. We define it as an episode of patient-clinician communication. Examples include a video call or a text exchange, which could occur over several days. We avoid the narrower terms “consultation” and “visit”, which can imply referral and reimbursement respectively. In one study, only 22% of interactions were reimbursed as visits^11^.

Next, a channel is a conduit for an interaction. Channels should be described as fully as possible, including who will participate (e.g., next available physician vs. a specific one), over what time frame, and through which modality. An example would be messaging with a nurse asynchronously by default and live should the nurse be available; with a guaranteed response time within defined hours; and with read receipts, typing notifications, and photo sharing. The lack of scheduling features can lead physicians to ask for them^6,12^ and may explain why certain initiatives have reported low uptake of video^6,13^.

Finally, we propose two primary outcomes: savings and likelihood of resolution. Savings are the cost difference, for all stakeholders including patients (e.g., travel costs) and employers (time away from work), between the virtual channel and the face-to-face alternative. Resolution consists of not only meeting the standard of care, but also having a good experience of care and leaving the patient with a sense of being cared for.

Maximizing these outcomes requires directing each interaction to the best channel, based on patient, problem and clinician characteristics. Marketers call this channel management. It relies on detailed analysis of touchpoints, conversion (goal attainment), cost structure and return on investment. With some adaptations, such as recommending channels rather than imposing them, channel management can be a keystone of value-based healthcare. A starting point for this practice is to understand the use of different channels and their potential impact.

## Virtual channels in primary care

Consider primary care as an example (see Table 1 for a summary of providers cited as examples). In the U.S. and Canada, regulation has favored video. Still today, only 11 state Medicaid programs reimburse store-and-forward technologies (transmission of text, images, and other media asynchronously), but all 50 cover video^14^. The Canadian province of British Columbia defines virtual visits as video^15^, so video visits and follow-ups are the focus of providers there, including UK-based Babylon Health. Another Canadian example is Dialogue Health, which offers virtual care purely as an employee benefit and thus lacks a reimbursement incentive to use video. The initial step here is triage over live chat with a nurse, who in 70% of cases resolves the issue^16^. Surprisingly, the remaining 30% are always handled via a video visit scheduled with a nurse practitioner or a physician. It may be that Dialogue chose to preserve synchrony and traditional workflows as a realistic first step to recruit clinicians and manage quality. Or perhaps the company believes that patients and clinicians interacting for the first time virtually prefer video.

**Table 1:** Selected virtual primary care providers and their approaches (alphabetical order).

Sample provider	Geography	Virtual modalities	Choice of clinician^a^	Scheduling^b^
98point6	USA	Chat^c^	None	No
AskMyGP	UK	Form → phone or asynchronous messaging	Possible	No
Babylon	UK, Canada, Rwanda	(Chatbot → ) video or audio	Possible	Yes
Dialogue^d^	Canada	Chat with nurse → video	None	Yes
Ping An Good Doctor	China	Chatbot → chat or video	None	No
Sherpaa^d^	USA	Asynchronous messaging	None	No
Teladoc	Global	Video, phone, chat	None	No

Sources: provider websites.

^a^No choice typically means the next available physician. The degree of choice varies across providers. Practices using AskMyGP may let patients choose their own physician or another physician from the same practice. Babylon offers a choice only when booking by phone. Sherpaa seems to offer no choice, but to have the same physician follow a patient between interactions of the same episode (and an episode may last several months, as in the case of breast cancer).

^b^Scheduling refers to booking a later appointment. No scheduling means that the visit occurs within minutes or hours of the patient requesting it.

^c^Chat refers to synchronous text-based communication with a clinician.

^d^Available within group plans only, e.g., via employers or insurers.

Of note, Babylon and Dialogue are among several companies investing in a new kind of channel: chatbots. Patients converse with these artificial intelligence (AI) engines by answering questions with free text or multiple choices. The Chinese service Ping An Good Doctor is betting on AI to scale up: among other strategies, it aims to expand its kiosks where patients chat with a bot, then have a virtual consult, and can collect common medications from an adjacent vending machine^17^. 98point6, a startup offering chat visits (like rivals CirrusMD and K Health), estimates that their bot currently conducts half of the initial interview with the patient^18^. Not all physicians will be interested in bot-elicited data, let alone bot diagnoses, but potential gains are clear insofar as AI can safely triage a growing proportion of queries to self-management^19^. Without a self-management option, a scenario observed in American telehealth data could become generalized: better access could merely tap into unmet demand, increasing both utilization and spending^20^.

While video and chat prevail in digital-first initiatives, two older channels seem more common overall: phone calls and asynchronous messaging. In England, phone calls are common^21^, notably on AskMyGP.uk—a service that handles requests from a practice’s rostered patients. On AskMyGP, the phone is the most requested and used modality, while asynchronous messaging ranks second^13^. Younger patients follow a similar trend: at Stanford in 2015, a clinic designed for 18- to 40-year old saw 37% of visits conducted by phone, 23% by video^22^. After all, while video may be superior for reassurance or rapport^23^, the phone suffices for many simple problems.

The other old modality is asynchronous messaging, through email or a dedicated platform. Many argue that messaging could bring radical change, if we redesigned workflows around it. Sherpaa, a startup acquired this year by the primary care provider for Apple and Facebook, focuses on this channel. Its CEO extolls the benefits of asynchrony: it gives clinicians time to look up information, allows reassessing over time, and is ideal for quick follow-ups like a forgotten question or a prescription renewal^24^. Indeed, physicians seem to favor asynchrony: in one pilot, physicians chose asynchronous messaging more often than patients did^6^. At Kaiser Permanente, the average physician exchanges 1217 emails with patients each year (but completes only eight video visits)^25^. For some conditions, patients may share information and engage more easily via messaging^26^. Savings could be significant: Mayo Clinic found that at least 40% of e-visits obviate in-person visits and that 80% require no further interaction. In particular, asynchronous follow-ups for chronic diseases appear to work well^27,28^. However, as with video and phone calls, there may be no gains or even net increases in workload if initiatives overlook workflow redesign or do not attain sufficient volume^29^.

A common simplifying strategy, employed by several of the abovementioned providers, is to focus on a single channel or sequence of channels. Multichannel strategies are more complex—retail banks, for example, execute them using dedicated teams and sophisticated software—but they are the natural next step to realize the full transformative potential of virtual care.

## The challenge of implementation

One challenge is to put in place high-quality channels. Providers and vendors should co-design channels together and look broadly for innovations, such as screensharing and co-browsing used in customer support. They should educate clinicians about channels and workflow redesign. Providers should orchestrate channel integration: for example, a video visit could be followed by an automated email check-in.

Another challenge is modeling and incentives. Health systems must build the capacities to predict likelihood of resolution and savings for each interaction in each channel and to incentivize users to choose the optimal channel. This requires collecting granular data and investing not only in marketing and reimbursement reform, but also in decision-support and analytics technology. For example, a triage system could flag a patient as typically unresponsive by chat and recommend a phone interaction instead. A physician who rarely renews medications through messaging could be notified of the untapped potential.

Today’s virtual care services typically focus a single sequence of channels or let users select channels without guidance. We can do better. Multichannel approaches should help patients and clinicians choose—and improve—how they communicate. They should also aim to increase access for all, rather than exclude patients and exacerbate inequities. A day may come when artificial intelligence will enable sophisticated channel management in healthcare, but for now the challenge that health systems face is to create effective channels, then manage them efficiently to best meet the needs of patients.
